# Size, shape, and flexibility influence nanoparticle transport across brain endothelium under flow

**DOI:** 10.1002/btm2.10153

**Published:** 2019-12-26

**Authors:** Maksymilian Nowak, Tyler D. Brown, Adam Graham, Matthew E. Helgeson, Samir Mitragotri

**Affiliations:** ^1^ John A. Paulson School of Engineering and Applied Sciences Harvard University 29 Oxford St. Cambridge MA 02138; ^2^ Wyss Institute of Biologically Inspired Engineering Harvard University 3 Blackfan Circle Boston MA 02115; ^3^ Center for Nanoscale Systems Harvard University 11 Oxford St. Cambridge MA 02138; ^4^ Department of Chemical Engineering University of California, Santa Barbara Santa Barbara CA 93106

**Keywords:** BBB, endothelium, microfluidic, nanoparticles, neurological disorders

## Abstract

Nanoparticle‐based therapeutic formulations are being increasingly explored for the treatment of various ailments. Despite numerous advances, the success of nanoparticle‐based technologies in treating brain diseases has been limited. Translational hurdles of nanoparticle therapies are attributed primarily to their limited ability to cross the blood–brain barrier (BBB), which is one of the body's most exclusive barriers. Several efforts have been focused on developing affinity‐based agents and using them to increase nanoparticle accumulation at the brain endothelium. Very little is known about the role of fundamental physical parameters of nanoparticles such as size, shape, and flexibility in determining their interactions with and penetration across the BBB. Using a three‐dimensional human BBB microfluidic model (μHuB), we investigate the impact of these physical parameters on nanoparticle penetration across the BBB. To gain insights into the dependence of transport on nanoparticle properties, two separate parameters were measured: the number of nanoparticles that fully cross the BBB and the number that remain associated with the endothelium. Association of nanoparticles with the brain endothelium was substantially impacted by their physical characteristics. Hard particles associate more with the endothelium compared to soft particles, as do small particles compared to large particles, and spherical particles compared to rod‐shaped particles. Transport across the BBB also exhibited a dependence on nanoparticle properties. A nonmonotonic dependence on size was observed, where 200 nm particles exhibited higher BBB transport compared to 100 and 500 nm spheres. Rod‐shaped particles exhibited higher BBB transport when normalized by endothelial association and soft particles exhibited comparable transport to hard particles when normalized by endothelial association. Tuning nanoparticles' physical parameters could potentially enhance their ability to cross the BBB for therapeutic applications.

## 1 INTRODUCTION

The use of nanoparticles to deliver therapeutics for systemic disease treatment has increased progressively since the approval of the first liposomal formulation of doxorubicin, Doxil®, in 1995.^1^ The appeal of nanoparticles lies in their ability to offer benefits such as extended drug circulation times, protection of drugs from degradation, controlled drug release, improved drug targeting, and potential to respond to external stimuli.^2^ As more advanced nanoparticles are developed for therapeutic applications, the extent to which their biological performance can be improved by altering the physical or chemical properties has received significant attention.^3^, ^4^, ^5^ The impact of modulating physical properties such as size, shape, flexibility, or charge is especially important to consider because these are some of the most fundamental nanoparticle attributes and can be tuned to improve performance.

Size is among the most studied nanoparticle parameters. Nanoparticles with sizes below ~10 nm, for example, are rapidly filtered by the kidneys and as a result often demonstrate minimal improvements in circulation times compared to free drugs.^6^ Particles of various sizes, even of micron size, have been designed for therapeutic applications. However, particles with diameters of ~400 nm or larger are not actively considered for most delivery applications.^7^ Particles larger than ~200 nm are prone to increased clearance via the reticuloendothelial system in the liver or spleen.^8^ Modulation of nanoparticle size can also be used to achieve passive accumulation in tumor tissue via the enhanced permeability and retention (EPR) effect.^9^ EPR is a result of the leaky vasculature formed within the tumors, wherein particles less than ~200 nm preferentially extravasate into tumor tissues, but only smaller particles (<30 nm) are cleared easily, though the size range can vary substantially depending on the tumor type.^10^

The influence of nanoparticle shape on biological performance has been extensively investigated. Cylindrical filomicelles have been shown to circulate in vivo for substantially longer times than comparable spherical vesicles.^11^ Rod‐shaped nanoparticles coated with targeting ligands have demonstrated increased target specificity in vitro compared to their spherical counterparts^12^ as well as improved organ targeting in vivo.^13^ Uncoated silicon‐based particles showed distinct shape‐dependent biodistribution upon intravenous injection, with discoidal particles accumulating significantly more in the lungs compared to their spherical, cylindrical, and hemispherical counterparts, whereas liver accumulation was much higher for cylindrical particles compared to the rest.^14^ Shape has also been implicated in altering cell‐specific responses to nanoparticles, especially uptake,^15^, ^16^ but also viability^17^ and cytoskeletal organization.^18^

Interest in assessing the impact of particle elasticity on nanoparticle delivery is a relatively recent development.^19^ Spherical poly(ethylene glycol) diacrylate (PEGDA) nanoparticles of different stiffness showed marked differences in circulation half‐lives, as well as uptake in macrophages, endothelial, and epithelial cells.^20^ Manipulating particle elasticity also has the ability to alter some of the classical trends observed with other properties, such as size. Hard microparticles are quickly cleared from circulation by the liver and spleen, but soft, discoidal microparticles (6 μm diameter) can circulate for days, and their circulation time is stiffness‐dependant.^21^ Soft discoidal particles also accumulate in tumors more than their stiff counterparts, though this is attributed primarily to the differences in circulation times of the particles.^22^

Delivery of nanoparticles to the brain is of particular interest because of the substantial burden that central nervous system (CNS) diseases place on society both in terms of lives lost and disability caused in nonfatal cases.^23^ The estimated cost of neurological diseases in the United States alone is over $800 billion.^24^ One of the major issues in treating brain diseases is crossing the blood–brain barrier (BBB), perhaps the body's most exclusive transport barrier. The BBB is notoriously difficult to cross; the microvascular endothelial cells that form the neurovascular unit are characterized by several restrictive features, including the expression of tight junction complexes, a lack of fenestrations, and low pinocytic activity.^25^, ^26^, ^27^, ^28^ As a result, there has been significant research into methods for improving nanoparticle transport through the BBB, using ideas ranging from physically disrupting the endothelium using ultrasound combined with microbubbles,^29^ to coating nanoparticles with ligands that enhance transcytosis,^30^ to using live cells as delivery vehicles.^31^ While there has been some investigation into how nanoparticle properties modulate transport into the brain,^30^ overall very little is known about how these differences affect their biological performance.

Improving the design of nanoparticles for brain delivery requires more information on how transport is affected by nanoparticle properties, and the utility of traditional in vivo and in vitro methods is limited in this respect. While in vivo experiments represent the gold standard because they incorporate all relevant aspects of an intact BBB and clearance mechanisms, deconvoluting the various biological contributions to transport and developing a mechanistic understanding for why certain attributes perform better is difficult. Moreover, methods such as capillary depletion may be inadequate for accurately and quantitatively assessing where particles accumulate,^32^ and measuring cargo delivery or using functional assays as a proxy for delivery of nanoparticles into the brain parenchyma provides an incomplete picture upon which to base future designs. These details are important because nanoparticle uptake by the brain endothelium is closely related to transport through it, but still a distinct process, as several reports have shown.^30^, ^33^, ^34^ Traditional in vitro approaches provide relatively simple and flexible platforms,^35^, ^36^, ^37^ but can suffer from model‐induced artifacts^38^ and limited temporal resolution. Moreover, they cannot incorporate hemodynamic shear stress, which has been shown to contribute substantially to realistic endothelial cell phenotype.^39^, ^40^, ^41^, ^42^ Development of dynamic microfluidic‐based in vitro systems that incorporate hemodynamic shear addresses several of these existing limitations.^43^ One such model particularly well suited to our investigations is the μHuB, a simplified and easy‐to‐use tool with real‐time imaging capabilities.^44^ The μHuB consists of a commercially available microfluidic chip scaffold and an immortalized cell line that incorporates physiologically relevant applied shear stresses and demonstrates size‐selective permeability to dextran tracers as well as expression of phenotypical tight junction markers. Here we report the use of the μHuB to study the impact of nanoparticle physical parameters on their ability to cross the BBB.

## 2 RESULTS AND DISCUSSION

### 2.1 Preparation of μHuB

**Figure 1:**
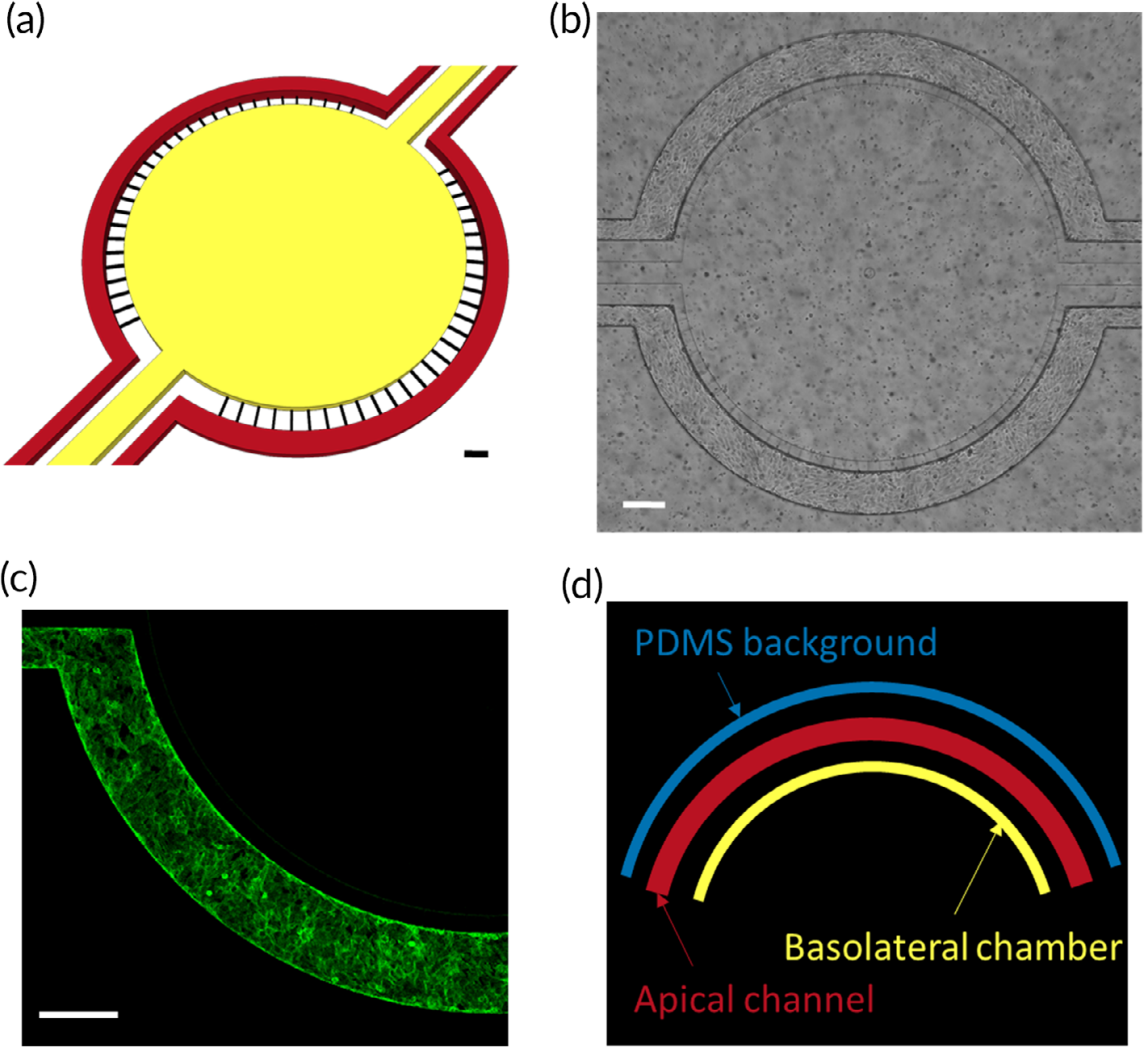
μHuB device. (a) Schematic of μHuB device shows apical channels (red) and basolateral compartment (yellow) as well as the pores that connect them (black) with appropriate dimensions. Scale bar = 200 μm. (b) Brightfield micrograph of μHuB device after flow conditioning. Scale bar = 200 μm. (c) Confocal slice of hCMEC/D3 monolayer in μHuB device stained with ActinGreen™ (green) indicates a complete monolayer. Scale bar = 200 μm. (d) Visual representation of the three measured regions of interest, the apical channel (center, orange), the BBB‐proximal basolateral chamber (bottom, green), and the BBB‐proximal PDMS region (top, blue)

The μHuB model was chosen for its optimal balance between being representative of the human BBB and being relatively simple to culture and use, as well as its ability to visualize transport in real time. hCMEC/D3 cells were used as a model cell line. While hCMEC/D3 cells are known for forming a relatively leaky barrier compared to primary cells, they are still considered one of the best model cells for the BBB.^45^ As the nanoparticles investigated herein (100 nm and larger) are too large to utilize a paracellular route through the BBB, this was not viewed as a major drawback. μHuB chips were prepared as described previously.^44^ After flow conditioning, hCMEC/D3 cells retained their preconditioning morphology, resisting elongation (Figure 1b) and formed a complete monolayer (Figure 1c).

### 2.2 Nanoparticle synthesis and characterization

Carboxylated polystyrene (PS) nanoparticles were chosen as a model particle due to their low toxicity in many biological systems,^46^, ^47^, ^48^ their negligible degradation over the timescales being studied,^49^ and their widespread use in research settings, which has resulted in thorough characterization and a diverse body of work to facilitate comparisons.^50^, ^51^, ^52^

**Figure 2:**
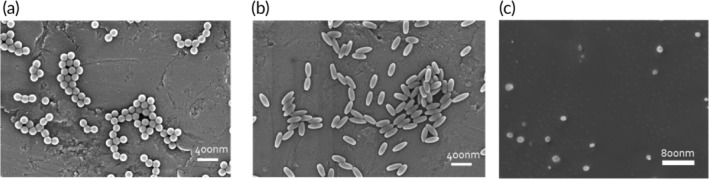
Nanoparticles of various sizes, shapes, and stiffnesses. Representative scanning electron micrographs of (a) 200 nm diameter polystyrene (PS) spheres, (b) 2AR PS rods, and (c) cryogenically prepared poly(ethylene glycol) diacrylate (PEGDA) spheres

To investigate the role of shape in crossing the BBB, rod‐shaped carboxylated PS particles were prepared via the film‐stretching method described previously.^53^ 200 nm diameter spherical particles were stretched to an aspect ratio of 2, meaning that particles are twice as long as they are wide (Figure 2b). The major and minor axes of these particles as measured by scanning electron microscopy (SEM) were 301.4 ± 15.7 and 120.2 ± 7.2 nm, respectively. The zeta potential of these rods was −39.0 ± 6.2 mV (Table 1).

**Table 1:** Summary of nanoparticle size, zeta potential, and modulus

Particle	*Z*‐average diameter (nm)	Polydispersity index (PDI)	ζ‐potential (mV)	Bulk modulus
100 nm PS sphere	120	0.01	−50.2 ± 8.3	3,000 MPa^54^
200 nm PS sphere	220	0.01	−47.4 ± 5.4	3,000 MPa^54^
500 nm PS sphere	590	0.14	−43.9 ± 5.5	3,000 MPa^54^
2AR PS rod	210	0.05	−39.0 ± 6.2	3,000 MPa^54^
PEGDA sphere	180	0.12	−34.1 ± 4.3	3 MPa^20^

Since PS nanoparticles are stiff, having a bulk modulus of approximately 3,000 MPa,^54^ we chose to synthesize softer hydrogel nanoparticles made with PEGDA that possess a bulk modulus of approximately 3 MPa.^20^ These nanoparticles have a similar size and zeta potential compared to 200 nm PS spheres (Table 1), and therefore are a good particle comparison to investigate the impact of particle elasticity on delivery through the BBB.

### 2.3 Nanoparticle toxicity on μHuB

**Figure 3:**
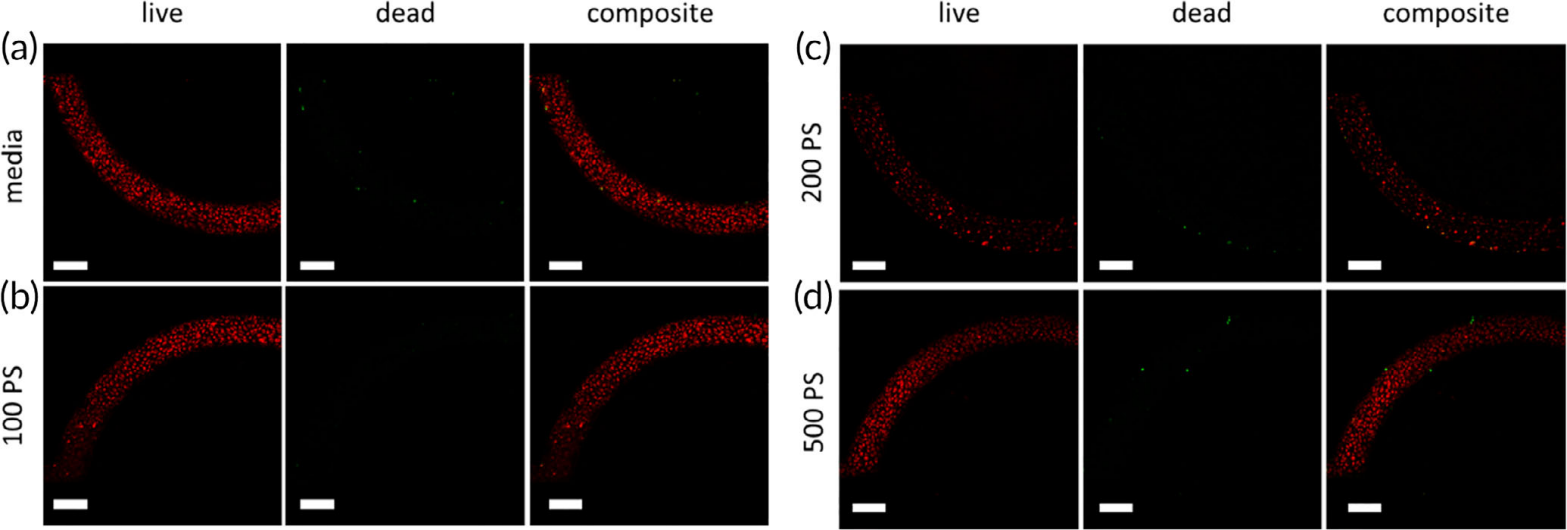
hCMEC/D3 monolayers remain viable after nanoparticle transport. Flow‐conditioned hCMEC/D3 monolayers show no appreciable difference in metabolic activity or cell death after 2 hr of flow with (a) media, (b) nonfluorescent 100 nm PS spheres, (c) nonfluorescent 200 nm PS spheres, and (d) nonfluorescent 500 nm PS spheres. All particle concentrations were identical to those used for endothelial association and transport experiments. Scale bars = 200 μm

The impact of nanoparticle flow on cell monolayer viability was assessed using a live‐dead assay. Cells with compromised cell membranes are stained with SYTOX Green, which is nonfluorescent until binding to the nucleus. Reduction of C_12_‐resazurin to red‐fluorescent C_12_‐resorufin is used as a measure of cell metabolic activity. hCMEC/D3 cells were grown in the microfluidic device, conditioned to shear stress, then kept under constant flow of cell culture medium for an additional 6 hr. These devices were then subjected to an additional 2 hr of constant flow with a nominal wall shear stress of 2.73 dyn/cm^2^ using a nonfluorescent nanoparticle solution to ensure particle fluorescence did not interfere with the viability assay. 100 and 200 nm spherical PS particles were flown at a concentration of 5 × 10^10^ particles/ml while 500 nm spherical PS particles were flown at a concentration of 2.3 × 10^9^ particles/ml, which is the same mass concentration as that for the 200 nm particles. Cells exhibited high viability and negligible cell death for all particle types compared to a media control (Figure 3) indicating that these particles are nontoxic to the monolayers at the concentrations used.

### 2.4 Endothelial adhesion, uptake, and transport of nanoparticles

Nanoparticle adhesion to the endothelium and uptake by endothelial cells under flow are key steps in transport cascade across the BBB. We therefore investigated the impact of nanoparticle physical parameters on these processes. μHuB devices were prepared as described, then imaged over time as a known concentration of each particle type was injected into the apical channel.

Fluorescence intensity in the apical channel arises from three different sources: particles flowing through the channel, particles that have adhered to the surface of the endothelium, and particles that have been taken up into the endothelial cells. As the flowing particle concentration remains constant, we are able to isolate the concentration of particles that are associated with the endothelium, either adhered to the cells or inside them. We report the concentration of nanoparticles associated with the endothelium after 2 hr, averaging the plateau concentration over time if the concentration clearly plateaus prior to the termination of the experiment. Any particles that accumulate in the basolateral chamber are considered to have transported through the BBB. Transport is reported as the rate of change in basolateral concentration over time.

### 2.5 Size‐dependent endothelial association and transport under flow

**Figure 4:**
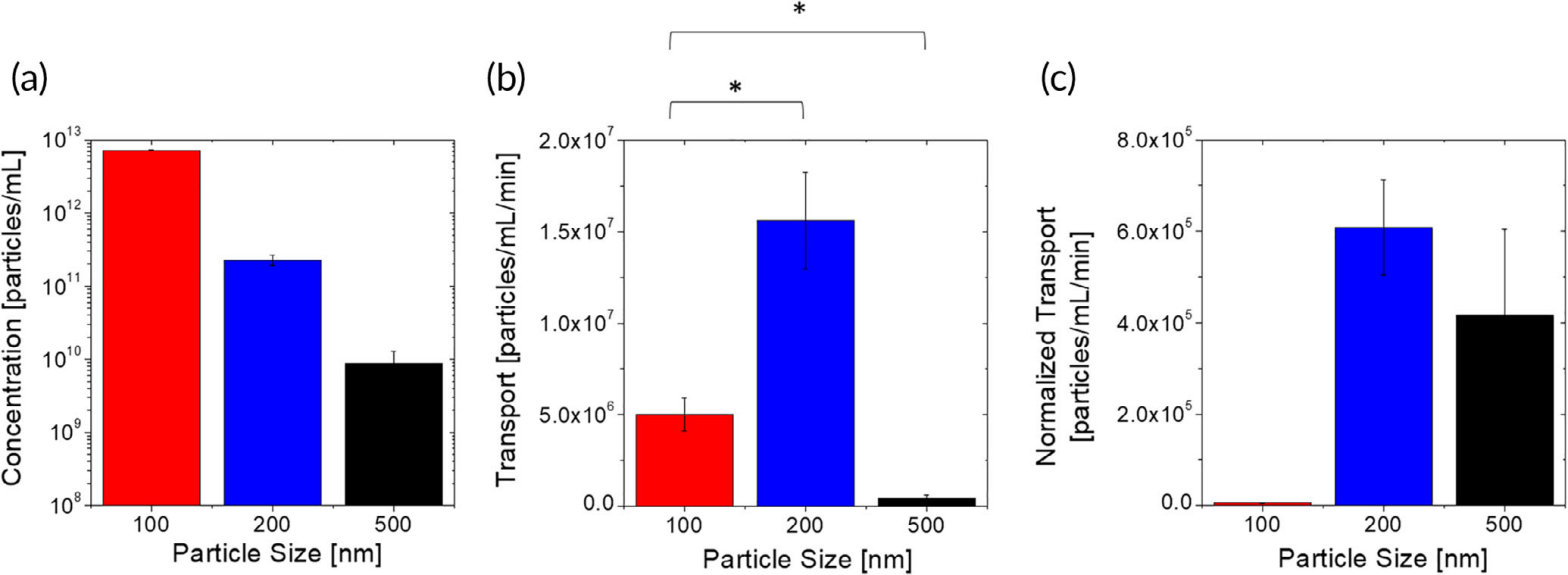
Size‐dependent nanoparticle–BBB interactions. (a) Endothelium‐associated concentration of spherical polystyrene nanoparticles of different sizes after 2 hr under flow. (b) Basolateral transport flux of spherical polystyrene nanoparticles of different sizes through the μHuB monolayer. (c) Basolateral transport flux of spherical polystyrene nanoparticles of different sizes through the μHuB monolayer normalized by the magnitude of their endothelial association

Endothelial association of particles exhibited a clear size‐dependence. One hundred nanometer PS spheres associate with the endothelium approximately 30‐fold more than 200 nm PS spheres when injected at an identical particle concentration (Figure 4a). Endothelial‐association of 500 nm PS spheres was 25‐fold lower than that of 200 nm PS spheres (Figure 4a). However, we note that 500 nm particles were injected at ~20 fold lower number concentration than 200 nm particles. When normalized for the injected concentration, 200 and 500 nm particles exhibited comparable endothelial association.

Three serial processes collectively impact the extent of particle association with the endothelium: margination from the flow to the wall, followed by binding to the cell membrane, then internalization by the endothelial cells. Previous studies on nanoparticle margination have shown that at a fixed injected particle mass, margination increases with increased size,^55^ indicating that the size‐dependence of particle association reported here is unlikely to be attributed to margination. This suggests that the observed trends on particle association arise collectively from binding and internalization. The relative contribution of the two, however, cannot be deconvoluted in this study.

The effect of particle size on adhesion to cellular or synthetic surfaces under flow has been investigated in quite some depth.^3^, ^56^, ^57^, ^58^ These studies have been generally performed with micron‐sized particles, thereby limiting comparisons to the present study. In one study, adhesion of spherical particles to surfaces under flow was reported and the dependence of adhesion on size was nonmonotonic with 500 nm particles exhibiting maximum adhesion compared to 100 nm, 200 nm, and 2 μm particles.^3^ Mathematical models have also been reported to describe wall adhesion of particles under flow. Modeling work by Tan et al. showed that 200 nm spherical particles exhibit lower binding probability to surfaces than 100 nm particles.^59^ Experiments examining particle adhesion to HUVEC cells under flow have also been conducted for particles as small as 500 nm.^58^ These experiments showed particle adhesion can be well described by a pure particle sedimentation model in linear laminar flow, with the number of adhered particles scaling like ~*d* ^−2^. Our observed trends on endothelial association do not exhibit a complete agreement with any of these previously reported trends. While this may arise from the differences in experimental systems, a likely reason is that cellular uptake represents a significant and potentially dominant component to measured endothelial association.

Studies explicitly investigating the separate contributions of cellular adhesion and uptake under flow have not been reported. However, Lin et al. have reported cellular association of PS nanoparticles with human aeortic endothelial under static and flow conditions.^60^ Under static conditions, a monotonic size‐dependence was observed, with 100 nm particles exhibiting the highest uptake and 1 μm particles exhibiting the lowest uptake. The size‐dependence of particle uptake under flow was not investigated in that study. The static uptake is in qualitative agreement with our monotonic size‐dependent endothelial association data, further suggesting that uptake represents a substantial component of endothelial association.

Size‐dependent transport rate data show a nonmonotonic trend, in which 200 nm spheres permeate most, with approximately threefold higher transport than 100 nm spheres. Five hundred nanometer spheres permeated significantly less, nearly 100‐fold lower transport than 200 nm spheres and approximately 10‐fold lower transport than 100 nm spheres (Figure 4b). This trend is preserved when normalizing the transport rate data by the amount of endothelial association (Figure 4c), and so necessarily indicates a nonmonotonic dependence of the rate at which internalized particles are transported through the BBB. The precise reasons for such nonmonotonic dependence are not clear. It is possible that particles of different sizes may follow different routes while permeating through the cell, though the specific nature of these differences will need to be investigated further. These transport data are in qualitative agreement with transport of silica nanoparticles through a static coculture rat BBB model, which showed that 100 nm spherical particles crossed more than 400 nm spherical particles, although transport for intermediate sizes was not reported.^61^ Literature data on the dependence of nanoparticle accumulation in the brain in vivo do not have sufficient granularity to quantitatively distinguish between endothelial accumulation and transport into the parenchyma.^62^, ^63^, ^64^ Further, these in vivo nanoparticle accumulation data are confounded by size‐dependent persistence of nanoparticles in the blood.

### 2.6 Shape‐dependent endothelial association and transport under flow

**Figure 5:**
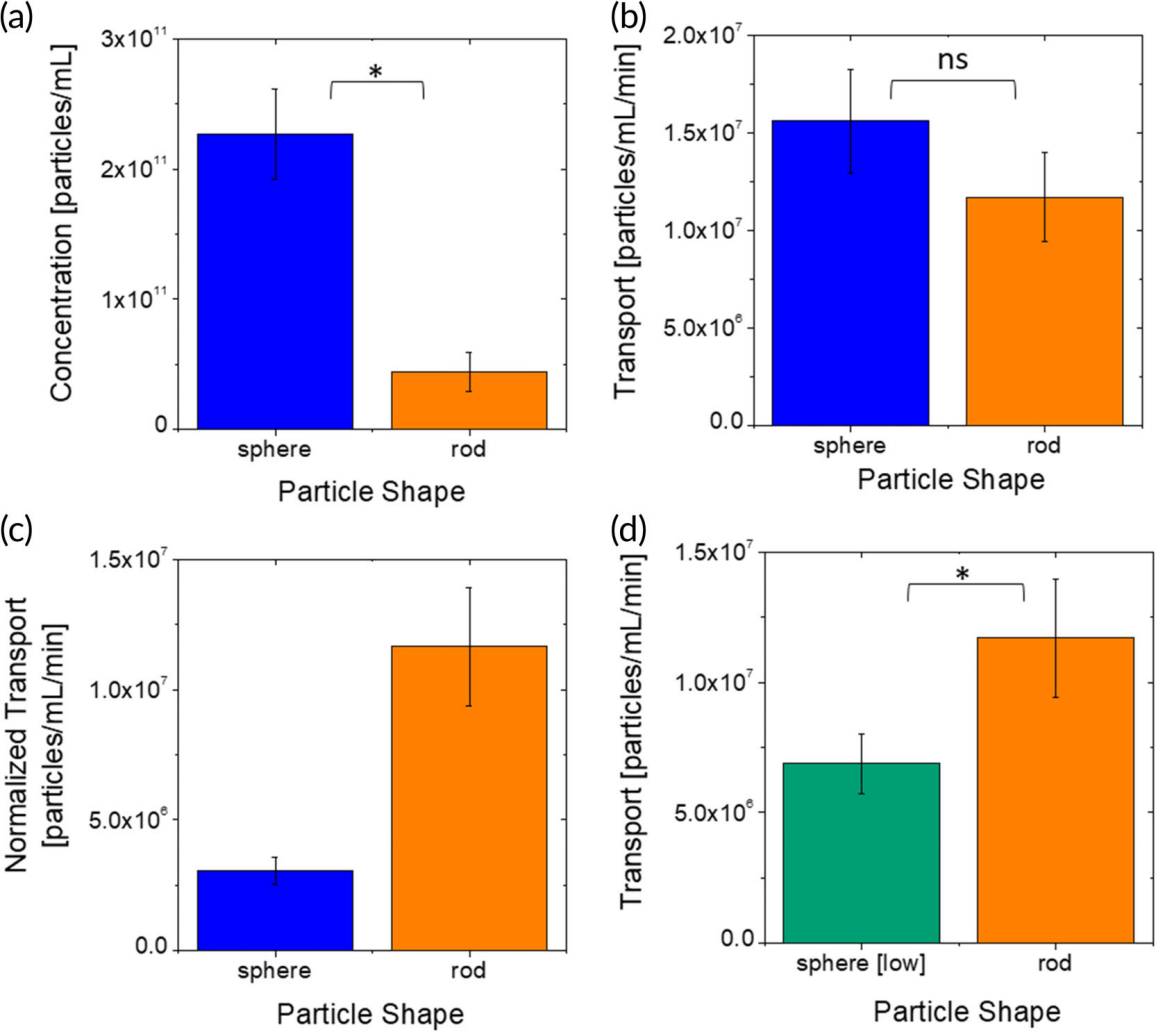
Shape‐dependent nanoparticle–BBB interactions. (a) Endothelium‐associated concentration of polystyrene nanoparticles of two shapes. (b) Basolateral transport flux of polystyrene nanoparticles of different shapes at the same concentration in solution through the μHuB monolayer. (c) Basolateral transport flux of polystyrene nanoparticles of two shapes through the μHuB monolayer normalized by the magnitude of their endothelial association. (d) Basolateral transport flux nanoparticles of two shapes when flow concentrations are adjusted to achieve equivalent endothelium‐associated concentrations of association in the μHuB monolayer

Shape‐dependent endothelial association data show that 200 nm PS spheres associate with the hCMEC/D3 monolayer about fivefold higher than rods of comparable volume (Figure 5a). Several studies have reported on the dependence of particle–cell interactions with shape in static cultures. These studies have yielded varying results. In general, the uptake of targeted rods is higher than that of spheres^13^, ^16^, ^65^ The dependence of uptake on nontargeted particles, on the other hand, has yielded different results. On synthetic surfaces, nontargeted rods have yielded lower adhesion compared to spheres. In contrast, studies with cell‐laden surfaces have shown that rods still exhibit higher association compared to spheres. Mathematical models have shown that the dependence of adhesion of particles on surfaces in turn depends on the interaction potential between the particle and adhering surface.^13^

The measured transport rate of rods and spheres across the endothelium was comparable (Figure 5b). When the trans‐endothelial transport is normalized by cellular association, the data demonstrate that rods exhibit significantly higher transport across the endothelium compared to spheres (Figure 5c). To further validate this conclusion, additional experiments were performed by using a lower concentration (1 × 10^10^ particles/ml) of spheres such that the total endothelial association of spheres and rods is comparable, thus eliminating the need to normalize for cellular association. Under this condition, the transport rate of rods is about twice that of spheres (Figure 5d) indicating that per unit particle that associates with the brain endothelium, rod‐shaped particles are better transported across the BBB compared to their spherical counterparts. We hypothesize that rods are trafficked through the cells using a route that is more efficient for transcytosis. Such differences could potentially involve fundamentally different pathways or simply enhanced efficiency of the same pathway for rod‐shaped particles. The precise nature of the differences in routes and their biological origins needs further investigation.

Effect of shape on in vivo accumulation of nanoparticles in the brain has been studied and these studies have yielded varying results. Using anti‐TfR antibody‐coated PS nanoparticles, Kolhar et al. reported increased accumulation of rods in the brain relative to their spherical counterparts after 6 hr.^13^ Similarly, Da Silva‐Candal et al. reported increased accumulation of anti‐VCAM antibody‐coated PS rods compared to spheres using a cerebral inflammation model.^66^ In another study comparing biodistribution of PEGylated gold nanospheres and nanorods in an orthotopic ovarian tumor model, brain accumulation (%ID/g) for spheres was higher than that for rods after 30 min.^67^ As with the size‐dependent comparison, these in vivo experiments do not have sufficient granularity to isolate the contributions of association versus transport or contributions from differences in circulation times in blood, preventing any direct comparison with the present study.

### 2.7 Stiffness

**Figure 6:**
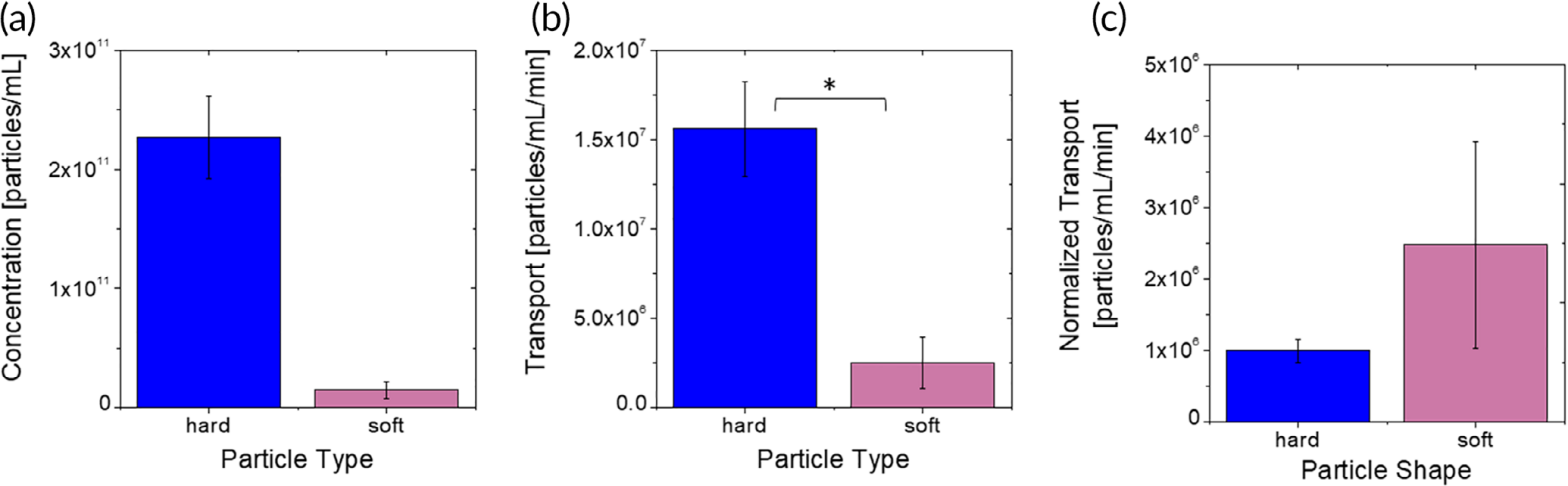
Stiffness‐dependent nanoparticle–BBB interactions. (a) Endothelium‐associated concentration of spherical 200 nm particles with different bulk moduli. (b) Basolateral transport flux of spherical 200 nm particles with different bulk moduli through the μHuB monolayer. (c) Basolateral transport flux of spherical 200 nm particles through the μHuB monolayer normalized by the magnitude of their endothelial association

The endothelium‐associated concentration of stiff 200 nm spheres made of PS was 10‐fold higher than their soft counterparts made of PEGDA (Figure 6a).

Endothelial association is significantly lower for PEGDA spheres compared to PS spheres. Under these conditions, both particle types have a small capillary number (Ca), which is the ratio of the viscous forces in the fluid and the elastic force in the solid.^68^ This indicates that the forces exerted by the flow are unlikely to appreciably deform either particle, and therefore any differences in adhesion are unlikely to be caused by differences in flow‐induced deformation.^69^ It should be noted, however, that as the particles are composed of different materials, it is possible that their adhesive interactions with various membrane components differ in ways that are not relevant to their mechanical properties despite their similar surface characteristics. PEG is often used to reduce adhesion of biological components to surfaces.^70^, ^71^, ^72^ Modeling on membrane wrapping of elastic nanoparticles shows that stiff particles are fully wrapped more easily than soft ones, indicating that cellular uptake is likely to be higher for stiff particles which is in agreement with our association data.^73^ Prior experiments examining the uptake of PEGDA nanoparticles with different stiffnesses in static culture over time showed that uptake was lower for softer particles regardless of cell type and presence of a targeting ligand.^20^ This suggests that endothelial association is primarily driven by uptake rather than adhesion for particles of different stiffness values.

Transport rates of soft spheres were about 10‐fold lower than those of hard spheres (Figure 6b). To our knowledge, the transport of nanoparticles with differing stiffnesses through a cellular monolayer has never been reported before. The data reported here show that nontargeted softer spherical particles exhibit lower transport than their hard counterparts, which is the same trend observed in endothelial association. When normalized by association, both hard and soft particles exhibited similar transport across the BBB (Figure 6c). We therefore hypothesize that differences in transport are driven primarily by differences in uptake. The impact of nanoparticle stiffness on brain accumulation in vivo is mostly unknown. Anselmo et al. reported that soft particles exhibit higher brain accumulation after intravenous injection of intracellular adhesion molecule (ICAM) targeted or nontargeted soft spheres.^20^ The difference between the softer and harder PEG particles was statistically significant, but relatively small and attributed to increased persistence of soft particles in circulation.

## 3 CONCLUSIONS

We have reported the ability of nanoparticles to associate with the brain endothelium and transport through it under flow and on the basis of particle size, shape, and flexibility. Each of these physical properties demonstrates a notable ability to tune nanoparticle interactions with the BBB. Importantly, our experiments highlight that endothelial association and basolateral transport are two coupled yet distinct processes, something bulk in vivo measurements cannot differentiate between. in vitro studies in the μHuB provide more granular data that are able to distinguish between these two mechanisms, and reveal that nanoparticle properties can differentially affect them. For example, whereas endothelial association of PS spheres increases with decreasing size, transport is optimal for 200 nm spheres, suggesting that 100 nm spheres accumulate on or within the endothelium. While PS rods and spheres have similar transport rates, rods associate with the endothelium significantly less. Stiff spheres both associate with endothelial cells and are transported through them much more than their soft counterparts. Understanding these distinct phenomena can be leveraged to develop better therapeutic nanoparticles for diseases in the endothelium, where transport into the brain is undesirable, but more importantly for parenchymal diseases where minimizing toxic effects on the vasculature without reducing transport would provide substantial benefits. Future investigations will identify the underlying mechanisms for these functional differences and test how these trends emerge for more clinically relevant nanocarriers. Depending on the nature of these mechanisms, it may be possible to apply similar approaches for optimizing the delivery of molecules or nanoparticles that utilize a specific receptor interaction to enter the brain.

## 4 MATERIALS AND METHODS

### 4.1 Particle synthesis and characterization

Spherical PS spheres were purchased from Polysciences and washed via centrifugation. PS rods were prepared via film‐stretching method.^53^ Briefly, particles were suspended in solution of polyvinyl alcohol and glycerol, then cast into a film. This film was then placed in a stretching apparatus and heated in an oil bath before being stretched to form rods. Stretched films were dissolved in water and particles were then washed via centrifugation. Particle morphology was assessed using SEM.

PEGDA nanoparticles were prepared by nanoemulsion templating method as described previously.^20^ Briefly, aqueous mixture of 40 vol% PEGDA, 1 vol% 2‐carboxyethyl acrylate, methacryloxyethyl thiocarbamoyl rhodamine B dye, and deionized (DI) water (1 ml) was emulsified in continuous phase of cyclohexane (15 ml) with Span 80 (300 mg) and Tween 80 (100 mg) surfactants on a stir plate. The emulsion was then ultrasonicated to form nanodroplets. After adding photoinitiator (2‐hydroxy‐2‐methylpropiophenone) nanoparticles were photo‐cross‐linked under UV light, then washed via centrifugation in cyclohexane. Particles were subsequently washed via centrifugation in water.

All nanoparticle concentrations were calculated by freeze‐drying a known volume of suspension and measuring the dry mass. Size and zeta potential measurements were obtained using a Malvern Zetasizer Nano ZS at room temperature.

### 4.2 μHuB preparation

hCMEC/D3 immortalized human cerebral microvascular endothelial cell line was purchased from Millipore Sigma and maintained using EndoGRO‐MV Complete Culture Media Kit supplemented with 1 ng/ml human animal‐free basic fibroblast growth factor (bFGF‐AF) and 1% Penicillin–Streptomycin. Tissue culture flasks were coated with 1:20 dilution of Corning® Collagen Type I, Rat Tail, at 37°C for 1 hr prior to use. Cells were incubated at 37°C, 95% humidity, and 5% CO_2_ until confluent. All cells used were between passages 28 and 35.

Idealized coculture microfluidic devices made up the scaffold for the μHuB and were purchased from SynVivo, Inc. (Huntsville, AL). Figure 1a shows the device schematic. Devices were prepared as previously described.^44^ Briefly, devices were coated with 300 μg/ml human fibronectin for 1 hr, then perfused and primed with nitrogen gas to remove bubbles. Cells were then injected at ~5 × 10^7^ cells/ml, the device was inverted, and cells were allowed to adhere to the upper polydimethylsiloxane (PDMS) surface. This process was then repeated with the device in an upright position. Cells were fed daily by perfusion of media. A linear ramping protocol (100 nl/min to 5 μl/min over 12 hr) was utilized to condition the cells to physiological shear stress.

### 4.3 μHuB lumen visualization

After flow conditioning the cells, device was perfused with DPBS to replace the cell culture media. 4% PFA was injected into all device compartments and incubated room temperature for 15 min. The device was then flushed with DPBS to move any residual PFA. Fixed cells were permeabilized using 0.2% Triton X‐100 in DPBS for 10 min at room temperature, then again perfused with DPBS to move any residual Triton X‐100. Thermofisher ActinGreen™ 488 ReadyProbes™ Reagent was used to stain for cytoskeleton, using two drops per milliliter of DPBS for 30 min at room temperature. The stain was replaced with DPBS prior to imaging.

### 4.4 μHuB viability analysis

LIVE/DEAD™ Cell Vitality Assay Kit, C12 Resazurin/SYTOX™ Green was used to assess cell viability after nanoparticle transport. Briefly, 10 nM of Sytox green and 500 nM of C_12_‐resazurin in DPBS was injected in the device. The device was incubated at 37°C, 5% CO_2_ for 15 min then imaged.

### 4.5 Quantification of nanoparticle association to and transport through μHuB

Nanoparticle experiments were conducted by flowing nanoparticle solutions (5 × 10^10^ particles/ml) through the apical channel at 5 μl/min for 2 hr. For 500 nm particles, a concentration of 2.3 × 10^9^ particles/ml was used instead due to toxicity and material considerations. This concentration is equivalent to 5 × 10^10^ particles/ml of 200 nm particles on a mass basis. Devices were kept at 37°C and 5% CO_2_ with a humidified Zeiss environmental enclosure. Images were acquired every 2 min using a 5X objective.

Raw images were corrected for variations in both shading and intensity.^74^ Corrected images were then imported into MATLAB and analyzed using a custom code. Briefly, the average pixel intensity and *SD* in each of the three relevant regions was calculated for each frame. These regions were the apical channel, the BBB‐proximal section of the basolateral chamber, and the BBB‐proximal section of the PDMS (Figure 1d). Because of the dramatic differences in intensity between the apical channel and the basolateral chamber, the PDMS region was necessary to correct for out of focus light intensity to ensure the measured output within the basolateral chamber is not convoluted with signal from the apical chamber.

After average region intensities were calculated, intensities were converted to physical particle concentration using calibration curves. Calibration curves were prepared by imaging known concentrations of each particle type in a straight microfluidic channel 100 μm tall, purchased from SynVivo, Inc. (Huntsville, AL). All calibration imaging conditions and corrections were kept consistent with μHuB live imaging conditions.

Endothelial cell association was determined by subtracting the particle concentration flowing in solution from the final concentration. For experiments where apical intensity clearly plateaued well before image acquisition was halted, the average value of the plateau intensity was used instead of the final intensity.

Transport was calculated by fitting a line to the concentration over time curve. Temporal or spatial regions where the local concentration increased at a physiologically improbable rate or to a physiologically improbable concentration were excluded. These events typically occur when the particle solution undergoes convective flow through the μHuB slits as a result of cells moving within the confluent monolayer.

### 4.6 Statistical analysis

Experiments were conducted at least in triplicate. Error bars represent the 95% confidence interval. Statistical significance was determined using a Student's *t* test with *p* = .05.
